# Micropropagation of *Opuntia* and Other Cacti Species Through Axillary Shoot Proliferation: A Comprehensive Review

**DOI:** 10.3389/fpls.2022.926653

**Published:** 2022-07-07

**Authors:** Sarah Bouzroud, Ezzouhra El Maaiden, Mansour Sobeh, Krishna Prasad Devkota, Hassan Boukcim, Lamfeddal Kouisni, Youssef El Kharrassi

**Affiliations:** ^1^African Sustainable Agriculture Research Institute (ASARI), Mohammed VI Polytechnic University (UM6P), Laâyoune, Morocco; ^2^AgroBioSciences Department (AgBS), Mohammed VI Polytechnic University (UM6P), Ben Guerir, Morocco

**Keywords:** areoles, Cactaceae, *in vitro* culture, multiplication, *Opuntia*

## Abstract

Cacti are one of the most significant and diversified groups of angiosperms, distributed and cultivated globally, mostly in semi-arid, arid, and the Mediterranean climate regions. Conventionally, they are propagated by seeds or through vegetative propagation *via* rooted offshoots or grafting. However, these multiplication procedures remain insufficient for mass propagation. *In vitro* culture techniques are utilized to mass propagate endangered and commercial cacti species. These include somatic embryogenesis and plant regeneration through indirect or direct organogenesis. The latter is a promising tool for commercial clonal propagation of high-value species and has been successfully implemented for several species, such as *Mammillaria, Hylocereus, Cereus, Echinocereus*, and *Ariocarpus*. However, its success depends on explant type, basal nutrient formulation of culture medium, and types and concentrations of plant growth regulators. This study aimed to assess the potential of *in vitro* propagation methods applied to cacti species and discuss the different factors affecting the success of these methods. This study has also highlighted the insufficient work on *Opuntia* species for mass propagation through axillary buds' proliferation. The development of an efficient micropropagation protocol is thus needed to meet the supply of increasing demand of *Opuntia* species for human consumption as fruit, animal feed, and ecological restoration in semi-arid and arid zones.

## Introduction

Cactaceae represents one of the most noticeable and diverse families of angiosperm available in nature and is cultivated mostly in warm and semi-arid and arid regions of the Americas, the Mediterranean countries, Africa, Asia, Europe, and Australia (Casas and Barbera, 2002). This family has nearly 130 genera, and around 2000 species present in different forms of growth and shapes such as columnar (*Carnegiea, Pachycereus, Stenocereus*, and *Lophocereus*), barrel (*Ferocactus* and *Echinocactus*), hedgehog (*Echinocereus*), pincushion (*Mammillaria*), cholla (*Opuntia*), and prickly pear (*Opuntia* and *Nopalea*) (Shedbalkar et al., 2010). Besides a remarkable diversity in growth forms, they are well-known for their great ability to grow and adapt under stressful environments, especially drought (Inglese et al., 2017; ICARDA, 2021). Cacti are often used to grow under marginal and sloppy lands as a barrier to soil erosion in West Asia (Jordan, Syria, Israel, and Yemen), Australia, and Europe (Italy, Portugal, and Spain), as a plant for ecological restoration and land reclamation, and also grown as a commercial crop such as fruit for human and feed for livestock (Khalafalla et al., 2007; Lema-Rumińska and Kulus, 2014; Inglese et al., 2017).

*Opuntia* is one of the well-known genera among the cacti, and *Opuntia ficus-indica* is the most important species cultivated for edible fruits and cladodes, vegetables, and animal feed, especially in the Mediterranean and the West and Central Asian countries (Glimn-Lacy and Kaufman, 2006; Shedbalkar et al., 2010; Nefzaoui et al., 2014; Inglese et al., 2017). Cacti are also known for their outstanding nutritional, medicinal, and biological properties conferred by the presence of high-value chemical compounds such as phenolic compounds, carotenoids, vitamins, flavonoids, and betalains (El-Mostafa et al., 2014; Díaz et al., 2017; Al-Khayri et al., 2018; González-Stuart and Rivera, 2019).

Cacti are propagated either from seeds or from vegetative cutting and grafting. Seed propagation is common and simple but difficult to obtain seedlings for some species due to the limitation of flowering, reproduction, and seed formation (Lema-Rumińska and Kulus, 2014). Cacti propagation through seeds is also time-consuming and requires specific light and temperature conditions (Monroy-Vázquez et al., 2017). Further, the use of seeds does not guarantee genetic stability and true-to-type progenies due to the predominance of cross-pollination and genetic diversity. In some cases, seed germination percentage is low due to the physical dormancy imposed by the hard coat. For *O. ficus*-*indica*, besides the low germination percentage (5.3%) recorded by Stambouli-Essassi et al. (2017), seeds evolved into cactus plants after 5 years of culture, while the first flowering can be achieved after seven years. In opposition to seed germination, conventional vegetative propagation is appropriate for the commercial production of cacti plants with high economical and agronomical values (Ghaffari et al., 2013). Even though vegetative propagation presents some advantages over seed germination, this method of propagation is mostly restricted to columnar species. The propagation rate can also be a limiting factor. The number of cladodes per plant has been estimated for vegetatively propagated cochineal carmine cactus pear forage-resistant species to be around 15.64 cladodes per plant after 540 days after planting (Lopes et al., 2013). Moreover, some cacti genera suffer from extreme extractivism, illegal trades and unsustainable harvesting, and the degradation of their natural habitat due to anthropic action (Jesus et al., 2019). These disturbances are largely responsible for the extinction threat of local populations and drastic changes in cacti distribution and abundance and can be exacerbated by slow growth and dependence on reproductive individuals for population maintenance (Hughes, 2017). Added to this, the limited number of plants produced using conventional techniques remains insufficient with the market need worldwide (Lema-Rumińska and Kulus, 2014). These difficulties related to market need supplying can partly be solved through the adoption of plant tissue culture techniques. Micropropagation is a powerful tool that allows the conservation of endangered species and mass-scale production of commercial varieties and species. For instance, through micropropagation, nearly 457,000 plants of *O. ficus-indica* can be produced from a single explant in a year (Rodriguez and Ramirez-Pantoja, 2020). However, this cannot be achieved without a better understanding of the different factors controlling the plant regeneration process in order to increase the efficiency of micropropagation techniques aiming the production and multiplication of superior genotypes of interest. This comprehensive review was carried out to assess the potential, advantages, and limitations of the available *in vitro* culture techniques applied for cacti micropropagation. Further, axillary buds' proliferation (the most important and specific method of *in vitro* culture), a promising *Opuntia* micropropagation technique, allows the development of new shoots and plants from preexisting meristems. A case study on micropropagation through axillary buds' proliferation, especially on the key parameters affecting its success, is required as it is one of the most rapid and reliable methods for *in vitro* clonal propagation and ensures the genetic homogeneity, epigenetic stability, and the production of true-to-type progenies.

## Methodology of Research

**Figure 1:**
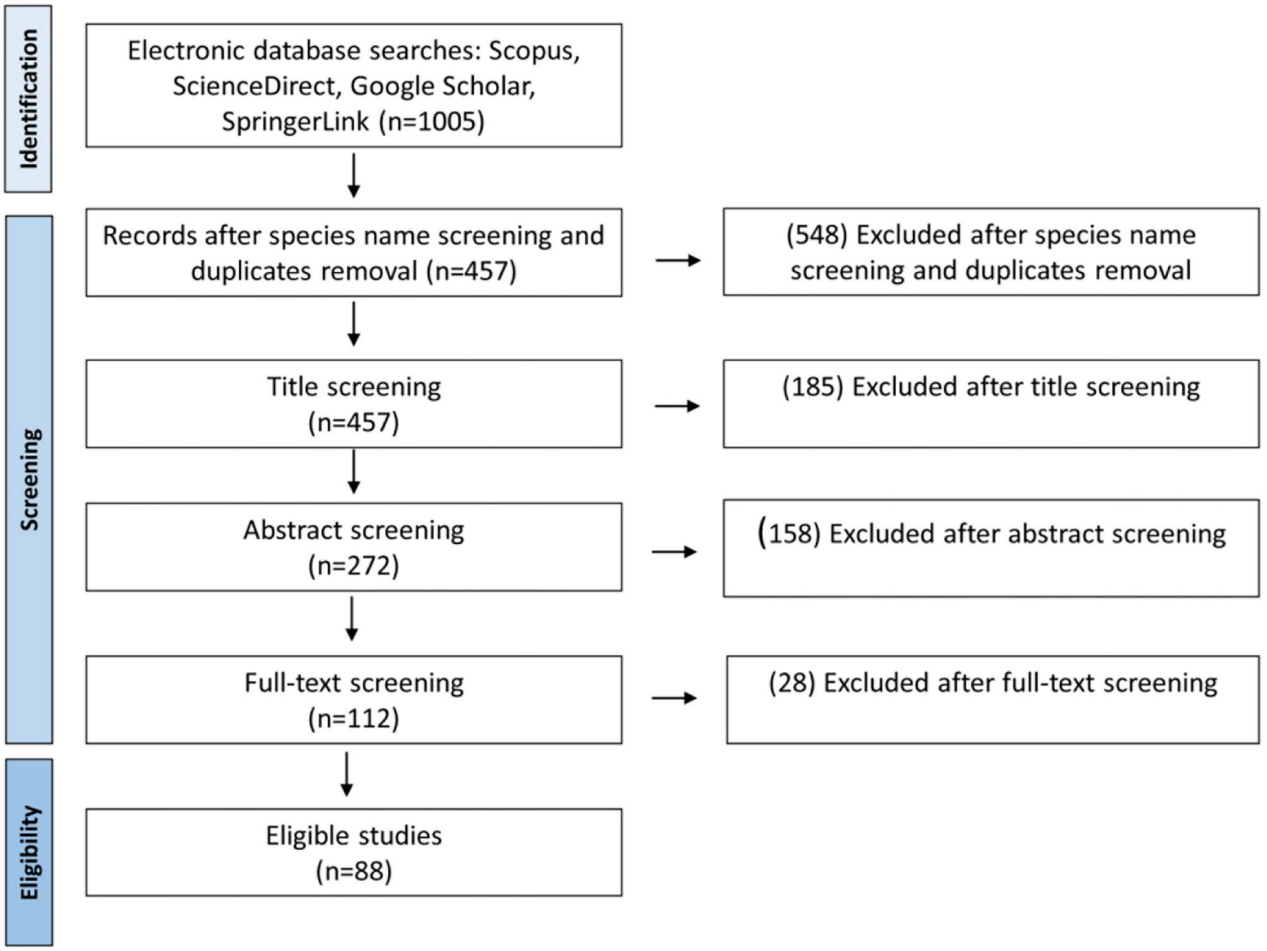
Literature search and selection process of the included studies of cacti and *Opuntia* species micropropagation.

For the review literature, a Web browsing was conducted in Scopus, ScienceDirect, Google Scholar, and Springer databases using keywords “cactus” or “cacti,” “propagation,” “*in vitro*” or “micropropagation,” “axillary buds,” or “areoles.” From the search, a total of 1,005 relevant studies were collected. From the collection, 457 duplicates were removed and 88 studies were preserved after screening the title, abstract, and full text. The outline of the literature search, database, and management is shown in Figure 1. Among the selected studies, “four” were about *in vitro* grafting, “eight” about somatic embryogenesis, and “49” related to regeneration through direct and indirect organogenesis.

## *In vitro* Propagation Techniques Applied to Cacti

**Figure 2:**
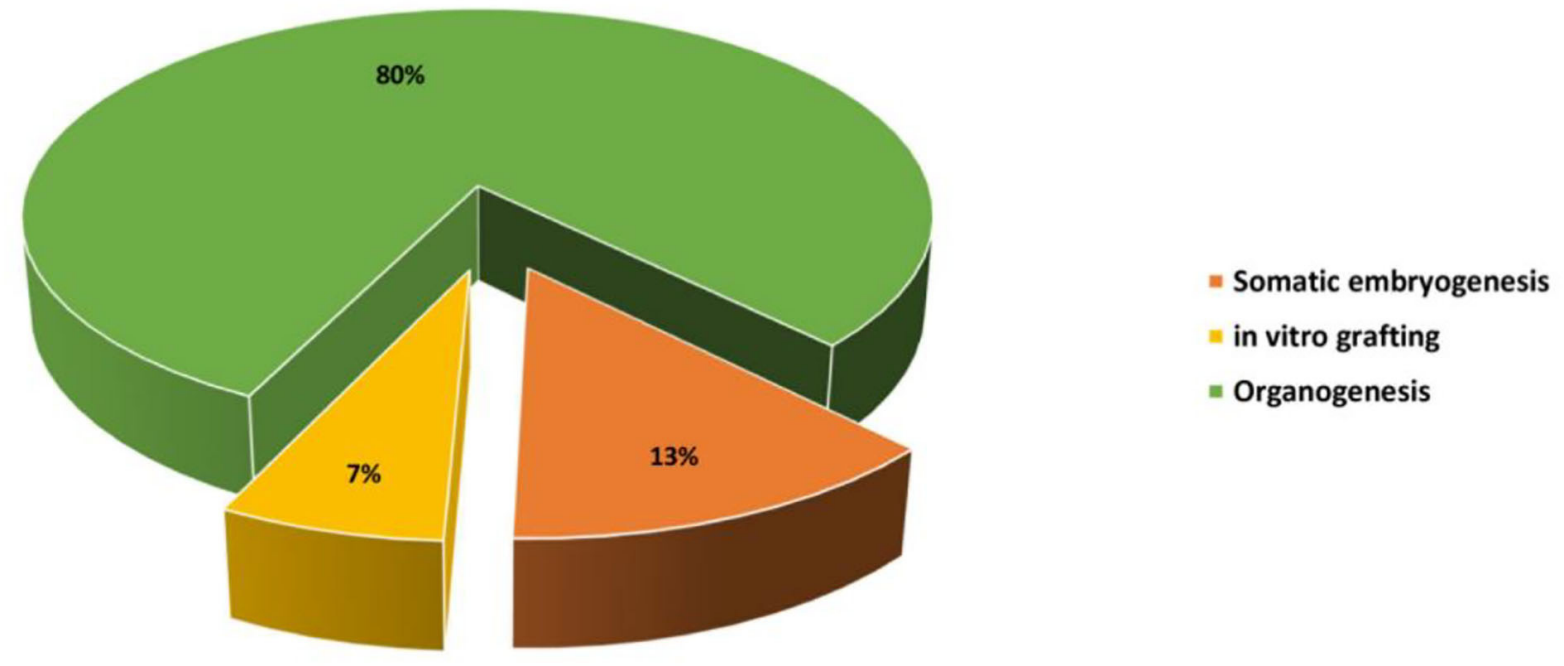
Percentage of the studies screened and reviewed for each method of *in vitro* propagation on cacti species.

**Figure 3:**
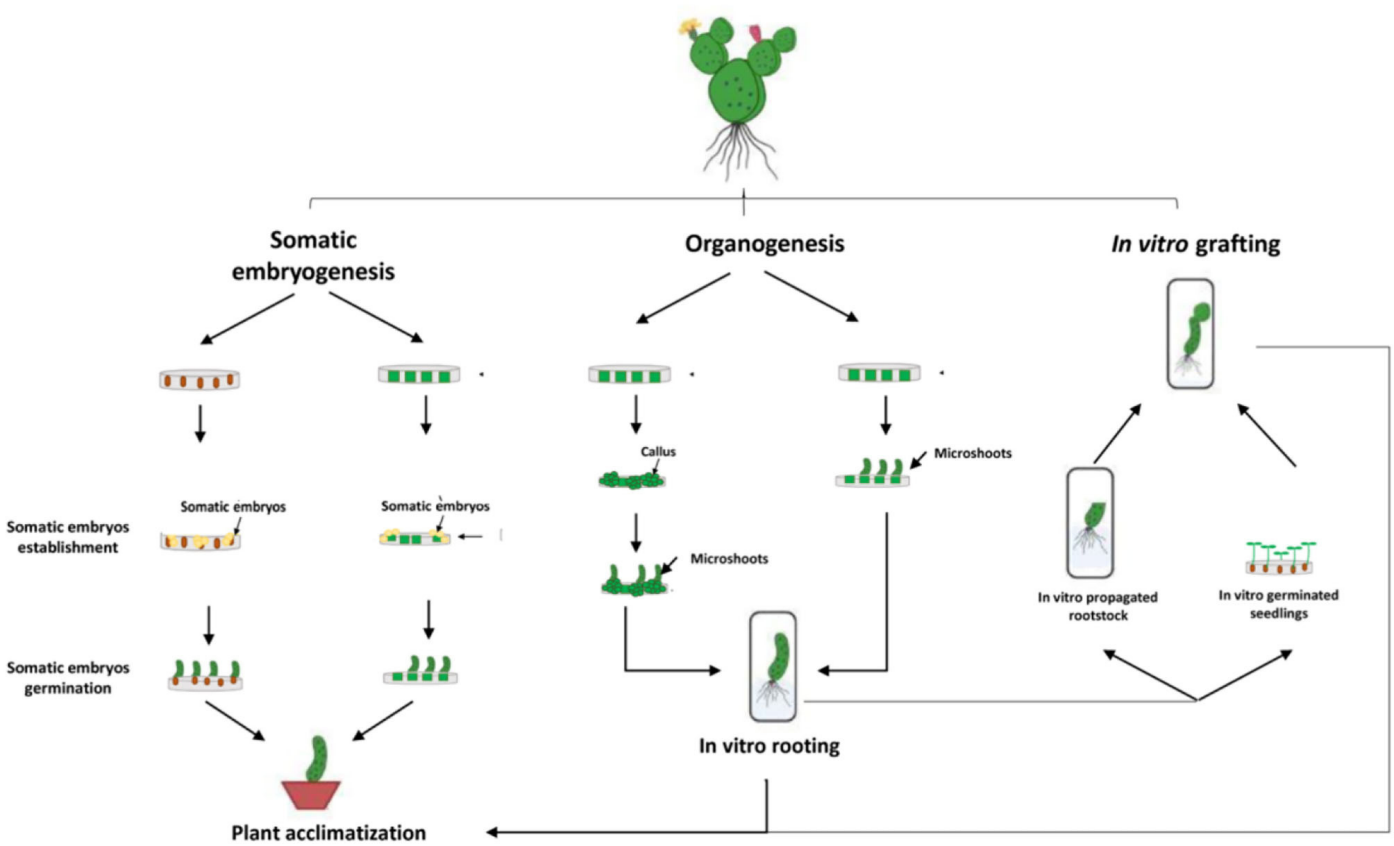
Overview of the different micropropagation techniques applied on cacti species.

Micropropagation can be defined as a vegetative method of propagation accomplished under aseptic *in vitro* conditions using small portions of the plant, also called explants, such as stem segments, cotyledons, leaves, axillary and apical buds, immature seeds, and embryos. Cactus micropropagation methods have been developed for over 60 years (Lema-Rumińska and Kulus, 2014). Numerous micropropagation techniques have been applied such as regeneration through somatic embryogenesis, regeneration through direct or indirect organogenesis, and *in vitro* grafting (Figure 2). Those methods have been used for several species of cactus such as *Mammillaria, Cereus, Hylocereus, Echinocereus, Astrophytum, Stenocactus, Schlumbergera, Coryphantha*, and *Copiapoa* (Wakhlu and Bhau, 2000; Papafotiou et al., 2001; Mohamed-Yasseen, 2002; Al-Ramamneh et al., 2006; Lema-Rumińska and Kulus, 2012; Lema-Rumińska et al., 2013; Bhau and Wakhlu, 2015; Chornobrov and Bilous, 2021; Ivannikov et al., 2021). The regeneration through direct or indirect organogenesis appeared to be the most important and reliable method of *in vitro* propagation with about 80% of the conducted work on cacti (Figure 3).

### Somatic Embryogenesis

Somatic embryogenesis is the process that allows the regeneration of whole plants from somatic embryos developed from somatic cells. Although these embryos are obtained from somatic cells, they show an identical pattern of embryo development commonly observed in zygotic embryogenesis (Gomes et al., 2006). Somatic embryogenesis has been applied in some cacti including prickly pear cactus (*O. ficus-indica*) (Gomes et al., 2006; Bouamama et al., 2011), *Ariocarpus kotschoubeyanus* (Moebius-Goldammer et al., 2003), *Schlumbergera truncata* (Al-Ramamneh et al., 2006), and *Astrophytum asterias* (Lema-Rumińska and Kulus, 2012).

In several cacti, somatic embryogenesis requires the presence of auxin or cytokinins alone. In *O. ficus-indica*, the addition of 4 mg/L of 4-amino 3,5,6-trichloropicolinic acid (picloram) to the semi-solid Murashige and Skoog (MS) basal medium allows the development of somatic embryos from shoot apices culture (Gomes et al., 2006). In *Astrophytum asterias*, somatic embryos derived from seeds were obtained using a higher concentration of 2,4-dichlorophenoxyacetic acid (2,4-D) (7 mg/L) (Lema-Rumińska and Kulus, 2012). Lema-Rumińska and Kulus (2014) succeeded in the induction of somatic embryos from areoles' culture of *Copiapoa tenuissima* by adding 2 mg/L of 2,4-D to the culture medium. For *Schlumbergera truncata*, somatic embryo culture was established from areoles' cultivation in a liquid Schenk and Hildebrandt (SH)-based medium supplemented with 1.5 mg/L of kinetin (Kin) (Al-Ramamneh et al., 2006). In some cases, somatic embryogenesis occurs as a result of the combined action of auxins and cytokinin. Tubercles of *in vitro* germinated seeds of *Ariocarpus kotschoubeyanus* evolved into somatic embryos in the presence of 6-benzylaminopurine (BA) (2–5 mg/L) and 1-naphthaleneaceticc acid (NAA) (0.1–1.1 mg/L) (Moebius-Goldammer et al., 2003). Indirect somatic embryogenesis can be initiated from immature anther culture in the presence of 2 mg/L 2,4-dichlorophenoxyacetic acid (2,4-D) and 2.5 mg/L of thidiazuron (TDZ) (Bouamama et al., 2011). MS basal medium supplementation with 5 mg/L of TDZ and 0.2 mg/L of NAA allowed the establishment of callus culture from Christmas cactus (*S. truncata*) axillary buds' culture and the proliferation of these callus cultures into somatic embryos (Chornobrov and Bilous, 2021). Somatic embryogenesis can also be trigged in the absence of auxin and cytokinins sources. Jedidi et al. (2013) were able to develop somatic embryos from immature ovule culture in a semi-solid MS medium supplemented with 1 mg/L of gibberellic acid (GA_3_).

### *In vitro* Grafting

*In vitro* grafting is the process of grafting a micro-scion onto the stems of young rootstock plants generated through *in vitro* culture (Ashrafzadeh, 2020). This propagation technique has been adopted for cacti as an alternative solution for the problems encountered during the propagation through grafting, mostly the poor success, contamination issues, and dehydration stress of graft union surrounding tissues (Estrada-Luna et al., 2002). Besides the development of pathogen-free plants, the scion growth requirements are directly supplied from the rootstock (Badalamenti et al., 2016). *In vitro* grafting has previously been tested for the genus *Opuntia*. Estrada-Luna et al. (2002) succeeded in the *in vitro* grafting of *O. ficus-indica* on five different rootstocks (*O. streptacantha, O. robusta, O. cochinera, O. leucotricha*, and *O. ficus-indica*). They also recorded the highest percentage of viability (up to 90%) with horizontal grafting, while only 30% of micro-grafted plants were able to survive when the scion was wedge-shaped (Estrada-Luna et al., 2002). *Gymnocalycium mihanovichii*, an ornamental cactus lacking chlorophyll, was successfully *in vitro* grafted on *Trichocereus spachianus*, with a 100% percentage of viability in the presence of indole-3-butyric acid (IBA) at 100 mg/L (Moghadam et al., 2012). *In vitro* grafting was found effective to ensure the propagation of endangered cacti species such as *Pelecyphora aselliformis* using *O. ficus-indica* as a rootstock (Badalamenti et al., 2016). *In vitro* grafting offers many advantages for the *in vitro* culture of cacti species, even endangered ones, which can contribute to the *ex vitro* conservation of threatened species (Badalamenti et al., 2016).

### Organogenesis

Organogenesis can occur directly from cells, tissues, or organs cultivated *in vitro* (direct organogenesis) or indirectly (indirect organogenesis) from a transitional step of callus formation (cell dedifferentiation) from which new plantlets are formed and developed from callus masses (Pérez-Molphe-Balch et al., 2015).

#### Indirect Organogenesis

Indirect organogenesis has been successfully implemented for several cacti species such as *Astrophytum asterias, Ariocarpus Kotschoubenyanus, Blossfeldia liliputiana, Strombocactus disciformis, Coryphantha elephantidens, Mammillaria elongata, Mammillaria gracilis, Mammillaria mathildae, Mammillaria pectinifera, Escobaria minima*, and *Pelecyphora aselliformis* (Wakhlu and Bhau, 2000; Papafotiou et al., 2001; Giusti et al., 2002; Poljuha et al., 2003; del Socorro Santos-Díaz et al., 2006; García-Rubio and Malda-Barrera, 2010; Ivannikov et al., 2021; Table 1). Callus-mediated regeneration commonly occurs when the culture medium is supplemented with high amounts of auxin or cytokinin during the initial step of callus formation (Pérez-Molphe-Balch et al., 2015). Newly formed buds are obtained after callus transfer to a culture medium with a high cytokinin/auxin ratio (Pérez-Molphe-Balch et al., 2015; Table 1). In Ariocarpus kotschoubeyanus, adventitious shoots have been obtained indirectly from tubercles (prominent cone-shaped protuberances, grooved on the upper surface and topped by an areole of spines) after 6 weeks of culture in the presence of BA alone or in combination with 1-naphthaleneacetic acid (NAA). The highest average numbers of buds per explant, that is, 6.2 and 6.3, were obtained with the application of BA (1 mg/L) and BA/NAA (3 mg/L BA and 1 mg/L NAA), underlining the efficiency of the technique. The highest shoot formation was also recorded within the same hormonal combination (Moebius-Goldammer et al., 2003). García-Rubio and Malda-Barrera (2010) established an efficient protocol of *Mammillaria mathildae* micropropagation through the culture of shoot basal segments in the presence of different concentrations of BA and indole-3-acetic acid (IAA). The cultivated explants developed callus on MS medium supplemented with different amounts of BA (0, 5, and 10 mg/L) and IAA (0, 0.25, 0.5, and 1 mg/L) (García-Rubio and Malda-Barrera, 2010). In *Mammillaria albicoma*, Wyka et al. (2006) obtained adventitious shoots from subsequent cultures of callus derived from flower buds culture in the presence of 0.1 mg/L of NAA and 5 mg/L of BA.

**Table 1:** Summary of the previous work related to indirect organogenesis of cacti species.

**Genus**	**Species**	**Explant type**	**Hormonal combination giving the best results**	**Number of shoots per explant/multiplication rate**	**References**
*Ariocarpus*	*A. kotschoubeyanus*	Tubercles	BA (1 mg/L) and BA/NAA (3 mg/L/1 mg/L)	6.2 with 1 mg/L of BA 6.3 with 3 mg/L of BA and 1 mg/L of NAA	Moebius-Goldammer et al., 2003
*Astrophytum*	*A. asterias*	Stem discs	1 mg/L BA	3.2	Ivannikov et al., 2021
*Blossfeldia*	*B. liliputiana*	Stem explants	1 mg/L BA	2.8	Ivannikov et al., 2021
*Coryphantha*	*C. elephantidens*	Tubercles	1.5 mg/L Kin and 0.5 mg/L 2,4-D	22.1	Wakhlu and Bhau, 2000
*Mammillaria*	*M. albicoma*	Flower buds	0.1 mg/L of NAA and 5 mg/L of BA	58 adventitious shoots from 3 flower buds	Wyka et al., 2006
	*M. gracilis*	N/A	No PGRs	N/A	Poljuha et al., 2003)
	*M. elongata*	Tubercles	mg/L BA and 0.1 mg/L NAA	10.3	Papafotiou et al., 2001
	*M. pectinifera*	Hypocotyl explants	1 mg/L TDZ.	32.2	Giusti et al., 2002
	*M. mathildae*	Basal explants	BA (5 mg/L)	0.18	García-Rubio and Malda-Barrera, 2010
*Notocactus*	*N. magnificus*	Hypocotyls	1 mg/L BA and 0.1 mg/L 2,4-D	6	de Medeiros et al., 2006
*Opuntia*	*O. ficus-indica*	Cladodes explants	0.5 mg/L 2,4-D and 0.5 mg/L BA	2.8	Angulo-Bejarano and Paredes-López, 2011
*Pelecyphora*	*P. aselliformis*	Hypocotyls	0.5 mg/L TDZ	85.5	Giusti et al., 2002
*Strombocactus disciformis*	*S. disciformis*	Stem explants	1 mg/L BA	3.7	Ivannikov et al., 2021

For cacti such as *Astrophytum asterias, Blossfeldia liliputiana*, and *Strombocactus disciformis*, the presence of auxin is not mandatory for indirect organogenesis. Indeed, the best organogenetic response was achieved in the absence of auxin (2,4-D) (Ivannikov et al., 2021). Kin or TDZ can also be used to induce indirect adventitious shoot formation instead of BA. Wakhlu and Bhau (2000) achieved *Coryphantha elephantidens* shoot regeneration through pith (storage tissue at the center of the vascular plant systems) tissue culture in MS medium enriched with 1.5 mg/L of Kin and 0.5 mg/L of 2,4-D. TDZ regenerative capacity was investigated by Giusti et al. (2002) for three different cacti: *Pelecyphora aselliformis, Escobaria minima*, and *Mammillaria pectinifera*. The authors found that the medium supplementation with 0.5 mg/L of TDZ prompted callus formation and then shoot regeneration of the three cacti species at a variable percentage (Giusti et al., 2002). Even though shoot can be regenerated from explant-derived callus, this method is less recommended than direct organogenesis, for clonal propagation due to the genetic instabilities favored by callus development. For instance, it has been previously reported that callus cultures or callogenesis promote somaclonal variation, which can contribute to genetic diversity (Sala et al., 2011). Therefore, indirect organogenesis is not recommended when aiming for the preservation of genetic integrity. However, it can be used in breeding programs, generating new varieties with new features, especially for ornamental, food, and medicinal purposes (Pérez-Molphe-Balch et al., 2015).

#### Direct Organogenesis

Direct organogenesis is defined as the direct process of the production of new buds from the tissue without intervening callus stage. During the last decades, micropropagation through direct organogenesis has been widely implemented to ensure the micropropagation of many endangered species, including those belonging to the Cactaceae family (Papafotiou et al., 2001; Pérez-Molphe-Balch and Dávila-Figueroa, 2002; Moebius-Goldammer et al., 2003; Dávila-Figueroa et al., 2005; de Medeiros et al., 2006; Ramirez-Malagon et al., 2007; Table 2). The success of this method of propagation relies mostly on the balance between cytokinin and auxin amounts. BA and NAA are commonly used to prompt direct shoot formation. For instance, an efficient protocol for direct organogenesis has been established for *Ariocarpus kotschoubeyanus* using BA alone or in combination with NAA. The highest number of buds forming explants was recorded in the presence of BA only (1, 3, and 5 mg/L) (Moebius-Goldammer et al., 2003).

**Table 2:** *In vitro* clonal propagation of cacti species *via* direct organogenesis reported in previous studies.

**Genus**	**Species**	**Explant type**	**Hormonal combination giving the best results**	**Number of shoots per explant/multiplication rate**	**References**
*Mammillaria*	*M. bocasana*	Shoot segments (at least 3 areoles)	10 mg/L Kin	3.8	Ramirez-Malagon et al., 2007
	*M. densispina*	Shoot segments (at least 3 areoles)	10 mg/L Kin and 4 mg/L IAA	4.8	Ramirez-Malagon et al., 2007
	*M. hahniana*	Shoot segments (at least 3 areoles)	10 mg/L Kin and 4 mg/L IAA	2.4	Ramirez-Malagon et al., 2007
	*M. hutchinsoniana*	Shoot segments (at least 3 areoles)	10 mg/L Kin	5.2	Ramirez-Malagon et al., 2007
	*M. orcutii*	Shoot segments (at least 3 areoles)	10 mg/L Kin and 1 mg/L IAA	17.4	Ramirez-Malagon et al., 2007
	*M. pectinifera*	Shoot segments (at least 3 areoles)	6 mg/L Kin and 4 mg/L IAA	4.5	Ramirez-Malagon et al., 2007
	*M. picta*	Shoot segments (at least 3 areoles)	10 mg/L Kin and 2 mg/L IAA	6.4	Ramirez-Malagon et al., 2007
	*M. perbella*	Shoot segments (at least 3 areoles)	10 mg/L Kin and 1 mg/L IAA	7.9	Ramirez-Malagon et al., 2007
	*M. rhodantha*	Shoot segments (at least 3 areoles)	6 mg/L Kin	5.4	Ramirez-Malagon et al., 2007
	*M. san-angelensis*	Shoot sections	6 mg/L IAA	26.77	Rubluo et al., 2002
	*M. zephyranthoides*	Shoot segments (at least 3 areoles)	6 mg/L Kin and 2 mg/L IAA	7.6	Ramirez-Malagon et al., 2007
*Micranthocereus*	*M. flaviflorus*	Cladode segments	0.25 mg/L NAA	6.4	Civatti et al., 2017a
	*M. polyanthus*	Cladode segments	1.5 mg/L Kin and 0.25 mg/L NAA	4.6	Civatti et al., 2017a
*Pelecyphora*	*P. aselliformis*	Areoles	2 mg/L BA	13.7 (first cycle) 128.1 (third cycle)	Pérez-Molphe-Balch and Dávila-Figueroa, 2002
	*P. strobiliformis*	Areoles	2 mg/L BA	12.4 (first cycle) 136.3 (third cycle)	Pérez-Molphe-Balch and Dávila-Figueroa, 2002
*Turbinicarpus*	*T. laui*	Shoot segments	1 mg/L BA	≈16	Dávila-Figueroa et al., 2005
	*T. flaviflorus*	Shoot segments	4 mg/L 2iP	≈14	Dávila-Figueroa et al., 2005
	*T. lophophoroides*	Shoot segments	2 mg/L BA	≈9	Dávila-Figueroa et al., 2005
	*T. pseudopectinatus*	Shoot segments	0.75 mg/ L BA	19.7	Dávila-Figueroa et al., 2005
	*T. schmiedickeanus*		2 mg/L BA	10	Dávila-Figueroa et al., 2005
	*T. subterraneus*	Shoot segments	1 mg/L BA	≈13	Dávila-Figueroa et al., 2005
	*T. valdezianus*	Shoot segments	2 mg/L 2iP	7.8	Dávila-Figueroa et al., 2005

In *Mammillaria* genus, culture medium supplementation with kinetin and IAA at different amounts stimulated adventitious shoot development (Ramirez-Malagon et al., 2007). The highest organogenic response was recorded in the presence of 10 mg/L of Kin for six different *Mammillaria* species (*M. hutchisoniana, M. picta, M. orcutii, M. bocasana, M. hahniana*, and *M. perbella*), while the lower concentration of kinetin (6 mg/L) was sufficient to induce the adventitious shoot formation for *M. rhodantha, M. densispina, M. zephyranthoides*, and *M. pectinifera* (Ramirez-Malagon et al., 2007). Nevertheless, the propagation rate was highly surpassed by combining IAA with Kin (4 mg/L IAA and 6 mg/L Kin) for this species (Ramirez-Malagon et al., 2007). However, other reports showed that the presence of cytokinin is not required for cacti *in vitro* propagation. It was previously underlined that adventitious shoot formation can also be stimulated in the absence of plant growth regulators or the presence of auxin alone (Papafotiou et al., 2001). In *M. elongata*, Papafotiou et al. (2001) recorded a better organogenic response in the absence of plant growth regulators or the presence of 0.1 mg/L of NAA. For *M. sanangelensis*, another member of the genus, a maximum proliferation rate was achieved in the presence of 6 mg/L of NAA (Rubluo et al., 2002). In *M. flaviflorus* and *M. densiflorus*, the best organogenic response was, however, observed in the presence of a lower NAA concentration (0.25 mg/L of NAA), which suggests that the presence of auxin is, in some cases, prerequisite for adventitious shoot induction (Civatti et al., 2017a,b).

## Constraints of *In Vitro* Propagation in Cacti

### Fungal and Bacterial Contamination

Cactus micropropagation is usually initiated by growing plants outdoors or under greenhouse conditions or from *in vitro* plants obtained from seeds (Lema-Rumińska and Kulus, 2014). Therefore, disinfection or surface sterilization of plant material (seeds or *in vivo* plant material) is mandatory to eliminate bacterial and fungal contamination present on the surface of plant material (Pérez-Molphe-Balch et al., 2015). Several disinfecting agents were applied for cacti surface sterilization, such as sodium hypochlorite (present in commercial bleach and other reagents), which has been widely used; however, its efficiency remains low. Combining sodium hypochlorite with ethanol appeared to be more effective to neutralize the exogenous microbiota of several cacti species; thus, this procedure has been widely employed (Estrada-Luna et al., 2008; Bhau and Wakhlu, 2015). In *Coryphantha elephantidens*, three-step disinfection was used for surface sterilization of young shoots, that is, thorough washing with tap water and 1% of detergent, a 1 min soaking in ethanol (70:30 v/v), and immersion in sodium hydrochloride for 15 min (Bhau and Wakhlu, 2015). The aseptic culture of *Opuntia robusta* cladodes was obtained by soaking in a biocide solution (comprising 3 mg/L benlate, 3 mg/L captan, 1 ml/L previcur, 0.5 g/L amoxicillin, and 0.4% of ketoconazole) before the application of hypochlorite solution. Within this disinfection protocol, the surface sterilization efficiency was nearby 90% (Astello-García et al., 2013). While using flowers as explants for micropropagation of *Mammillaria* genus (*M. albicoma, M. carmenae*, and *M. schiedeana*), Wyka et al. (2009) performed the surface sterilization of flowers by soaking them in 70% of ethanol for 1 min, followed by an active shaking in sodium hypochlorite solution (0.15% (v/w) available chlorine) for 5 min.

Besides sodium hypochlorite, other disinfecting agents, such as mercury chloride, have been tested in combination with alcohol for surface sterilization. For instance, Chornobrov and Bilous (2021) succeeded in the establishment of aseptic cultures from Christmas cactus (*S. truncata*) areoles (isolated from phylloclades), with more than 90% of decontaminated explants using a hydro-alcoholic solution of ethyl alcohol (70:30 v/v), followed by submersion of 0.1% of mercury chloride solution.

As an attempt to overcome the contamination issue encountered with the *in vitro* propagation of cacti using *in vivo* plant material, several authors initiated aseptic culture from *in vitro* germinated seeds. *O. ficus-indica* seeds were disinfected using 20% sodium hypochlorite combined with Tween 20 for 5–10 min, following a deep cleaning under running tap water (Heikal et al., 2021). Cortés-Olmos et al. (2018) used ethanol and sodium hypochlorite for *Lophophora williamsii* seeds' disinfection. For sweet cactus (*Nopalea cochenillifera*) species, the lowest contamination percentage was obtained when seeds were disinfected with 70% of ethanol for 1 min, followed by a deep soaking in a commercial bleach solution (sodium hypochlorite) at 1.5% for 10 min (Castro et al., 2011). Resende et al. (2021) applied a higher concentration of ethanol (96%) and sodium hypochlorite (2%) for the surface sterilization of *Melocactus glaucescens* seeds. The surface sterilization was established using 0.1% of commercial detergent (Extran) before the disinfection with 70% of ethanol for 1 and 25 min soaking in sodium hypochlorite solution at 2% (Dávila-Figueroa et al., 2005).

### Explant Necrosis

Explant necrosis resulting from the phenolic compounds' release is another potential problem that has been reported with cacti micropropagation (Lema-Rumińska and Kulus, 2014). The incidence of explant necrosis in *Mammillaria* genus as the result of the intensive and widespread oxidation has been explained by Ramirez-Malagon et al. (2007). Phenolic compound secretion can be prevented or neutralized by the addition of antioxidant substances in the culture medium (Lema-Rumińska and Kulus, 2014). Ascorbic acid, citric acid, active charcoal, and polyvinylpolypyrrolidone (PVP) have widely been used for this purpose. For instance, Lázaro-Castellanos et al. (2018) used activated charcoal at 1 g/L for the *in vitro* propagation of three *Mammillaria* species to reduce explant oxidation. Explant browning has been controlled in *H. undatus* callus cultures using activated charcoal at 1 g/L (Cruz and Balendres, 2021). Mabrouk et al. (2021) also added 1 g/L of activated charcoal to the culture medium for *Opuntia spp*. micropropagation. A higher concentration of activated charcoal, that is, 3 g/L, was used for the *in vitro* germination and shoot development of *Hamatocactus setispinus* (Xavier and Jasmim, 2015). A lower concentration of activated charcoal was used for pitaya cultures. Lee and Chang (2022) were able to neutralize phenolic compounds secreted during the *in vitro* culture of pitaya (dragon fruit) through the addition of 200 mg/L of activated charcoal. Tissue oxidation was partially prevented in *Nyctocereus serpentinus, Ferocactus*, and *Coryphantha* by the addition of 2 g/L of PVP (Balch et al., 1998). A combination of antioxidant substances is required in some cases. Non-oxidized callus cultures from *O. robusta* have been initiated using a combination of antioxidant substances amended at different concentrations, namely activated charcoal (10 g/L), ascorbic acid (0.025 mg/L), citric acid (0.025 mg/L), and PVP (3 g/L) (Astello-García et al., 2013). It appears that many cacti produced high amounts of phenolic compounds, which lead to explant oxidation. Culture medium supplementation with the appropriate amounts of antioxidant substances prevents oxidative stress as a result of the accumulation of phenolic compounds (Lema-Rumińska and Kulus, 2014).

### Hyperhydricity (Vitrification)

Hyperhydricity or vitrification is a morphological disorder occurring during the *in vitro* culture. In cacti, hyperhydrated plants showed an excessive accumulation of water within cells or tissues, low lignification in the inner part of the rind which enables cells to store high amounts of water causing cell enlargement, a signification reduction in photosynthetic performance and photosynthetic pigments, and impaired stomatal function resulting in a glassy transparent appearance and reduced mechanical strength (Balen et al., 2012; Borland et al., 2018; de Souza et al., 2019). Vitrified plants show a distinctive phenotype with a reduced number of spines and/or roots (Lema-Rumińska and Kulus, 2014). The vitrified shoots display structural alterations in chloroplasts and plastids such as chloroplasts dedifferentiation, thylakoid lump, plastid swelling, and phytoferritin accumulation (Balen et al., 2012; Lema-Rumińska and Kulus, 2014). As a result, hyperhydrated plants failed to survive in field conditions due to their reduced photosynthetic performances (Quiala et al., 2009; de Souza et al., 2019). This failure has been estimated in *T. valdezianus* up to 65% (Ht et al., 2011). This abnormal phenomenon, more likely observed in *in vitro* propagated plants, is seemingly linked with oxidative stress that occurs at the cellular level, as a result of stressful *in vitro* growth conditions such as light intensity and relative humidity, high amounts of plant growth regulators, and gas release by explants in the cultivation containers (such as petri dishes and jars) (Saher et al., 2004; Ht et al., 2011).

Hyperhydricity is a serious problem limiting *in vitro* propagation of many Cactaceae species (de Souza et al., 2019). de Souza et al. (2019) have reported a high percentage of hyperhydricity (88–100%) in *O. stricta in vitro* plants regenerated in the presence of BA. About 75% of hyperhydricity was obtained in *T. valdezianus* shoots regenerated through cladodes' segment culture in the presence of BA (Ht et al., 2011). Hyperhydrated shoots were also obtained while cultivating *Pelecyphora aselliformis* areoles in the presence of ~2 mg/L of BA (Santos-Díaz et al., 2003). A concentration of 3 mg/L of BA increased hyperhydration incidence in *Pilosocereus robinii in vitro* shoots (Quiala et al., 2009). In other cacti, in shoots regenerated through areoles culture, the incidence of hyperhydricity estimated was 64.3%, 71%, and 85.2% for *Mammillaria pectinifera, Escobaria manima*, and *Pelecyphora aselliformis*, respectively (Giusti et al., 2002). For *Micranthocereus*, Civatti et al. (2017a) reported any hyperhydrated shoots when explants were cultivated in half-strength MS/2 supplemented with various NAA and Kin concentrations.

Several attempts have been made to reduce the occurrence of hyperhydricity in cacti species. Santos-Díaz et al. (2022) have found that the hyperhydricity incidence can be minimized by decreasing the salt concentration of the medium (the use of quarter-strength MS medium for example). The reduction in salt concentration, more precisely ammonium (NH_4_)/nitrate (NO_3_) ratio, is also effective to decrease shoot hyperhydricity in *T. mombergeri*, and a similar finding was also reported in *Cereus jamacaru*. For instance, vitrified and abnormal shoots of *C. jamacaru* recovered with the reduction in basal salt, vitamins, and sugar concentrations (Monostori et al., 2010). The increase in calcium amount (double-dose) also prevented the hyperhydration of regenerated shoots (Santos-Díaz et al., 2022). This positive effect of calcium was previously explained by White and Broadley (2003) as the putative role of calcium in cell wall reinforcement. The high affinity of calcium is linked with the pectic chains, which provide additional rigidity to cell walls (White and Broadley, 2003). Calcium is an important element for cactus nutrition and a prerequisite for proper shoot development (Santos-Díaz et al., 2022). Vitrification can also be reduced using high amounts of gelling agents (ex. 10 g/L of Agar) that reduce water availability in the medium culture (Pérez-Molphe-Balch et al., 2002). Polyethylene glycol addition to the medium culture lowers shoot hyperhydration in several cacti species such as *A. kotschoubeyanus, T. laui, T. valdezianus*, and *P. aselliformis* (del Socorro Santos-Díaz et al., 2003, 2006; Ht et al., 2011). Besides polyethylene glycol, other chemical substances, such as gibberellic acid synthesis inhibitor: paclobutrazol, were successfully used for this purpose (Ht et al., 2011). Paclobutrazol reduces endogenous gibberellin production and results in the inhibition of cell elongation (Hedden, 2020). The use of paclobutrazol in *T. valdezianus* allowed the development of normal shoots without any hyperhydricity symptoms (Ht et al., 2011). Overall, minimizing the excess accumulation of water that occurs in the *in vitro* culture (due to high basal salt concentrations, high cytokinins, and endogenous plant hormone amounts along with high water availability) is an efficient way to minimize hyperhydricity incidence in cacti *in vitro* shoot development (de Souza et al., 2019).

## Micropropagation Through Axillary Buds' Proliferation in *Opuntia* Species: a Case of Study

**Figure 4:**
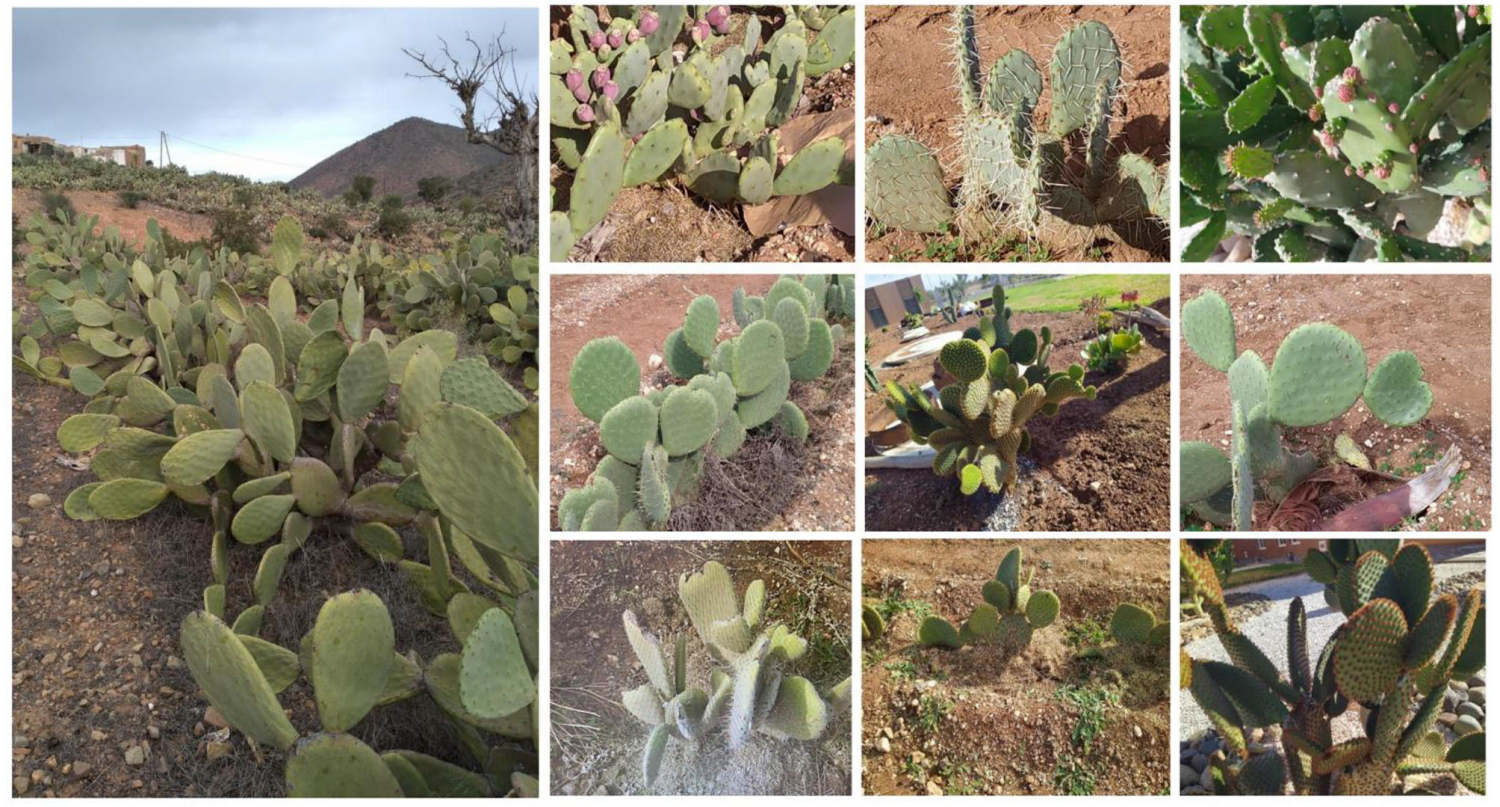
Diversity of *Opuntia* species present in Morocco.

Genus *Opuntia* belongs to the Cactaceae family, subfamily *Opuntioideae*, tribe *Opuntieae* (Omar et al., 2021). *Opuntia* genus comprises nearby 181 species and 10 naturally occurring hybrids (Anderson, 2001). The most widespread, cultivated, well-known, and economically important species that belong to this genus is *O. ficus-indica*. Besides *O. ficus-indica*, a few species such as *O. robusta, O. hyptiacantha, O. streptacantha, O. albicarpa, O. amyclaea, O. megacantha*, and *O. cochenillifera* are cultivated in different areas of the world (Omar et al., 2021). In semi-arid land of Morocco and other Mediterranean regions, several spiny and spineless *Opuntia* species are cultivated, especially for fruit production or animal feeding (e.g., *O. ficus*-*indica* and *O. stricta*) or for ornamental purposes (*O. macrocantha* and *O. microdasys*) (Figure 4). However, the production of many *Opuntia* species in nature is now at risk due to overexploitation, reduction in their natural areas, and cochineal infestation (Mabrouk et al., 2021). Thus, the establishment of reliable methods for mass propagation of prickly pear cultivated species is required to meet the high market demand and, ultimately, ensure the restoration and the rehabilitation of these species in their natural environment (Mabrouk et al., 2021).

**Figure 5:**
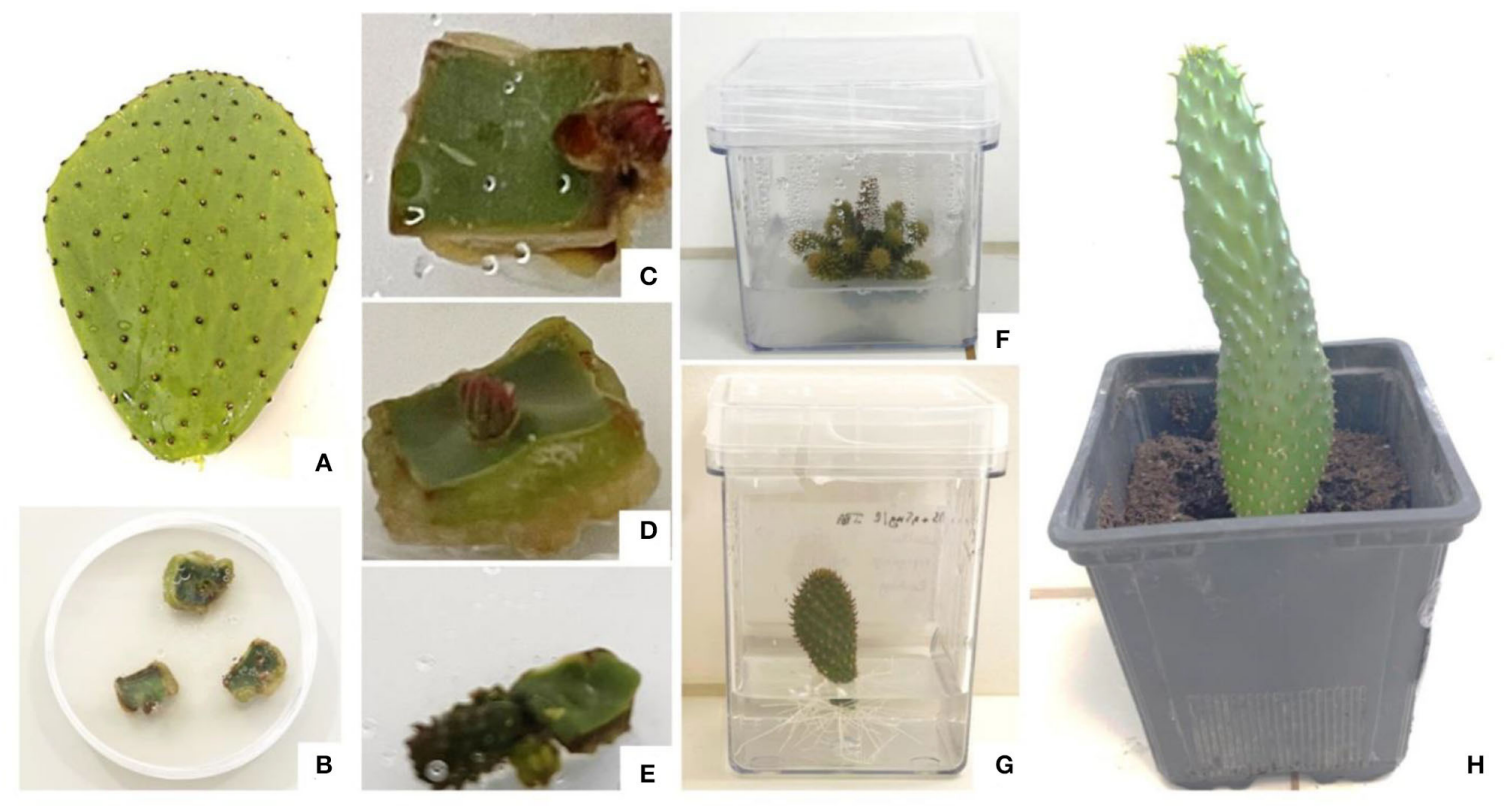
*In vitro* regeneration of *Opuntia spp* through axillary buds' proliferation. **(A)** *Opuntia spp* cladode collected from *in vivo* propagated plant, **(B)** areole isolation and aseptic culture establishment, **(C,D)** areole activation after 3 and 4 weeks of culture, respectively, **(E)** shoot initiation after 5 weeks of culture, **(F)** shoot multiplication from a single shoot, **(G)** *in vitro* rooting of *in vitro* shoots, and **(H)** acclimatization of *in vitro* regenerated plantlet in peat. The pictures were taken from micropropagation assays conducted at the African Sustainable Agriculture Research Institute, Mohammed VI Polytechnic University (ASARI-UM6P), Laâyoune, Morocco.

Micropropagation through axillary buds sprouting is widely used to ensure the genetic homogeneity and stability and produce true clones of plants. Axillary bud growth is mostly controlled by the shoot apex or apical dominance (Bredmose and Costes, 2017). Activation of apical dominance is triggered by the action of growth regulators such as cytokinins for areoles (axillary buds) and dormant axillary buds' sprouting and multiplication, and synergetic cytokinin and auxin combinations to prompt shoot multiplication (Ngezahayo and Liu, 2014). This process relies on the activation of dormant axillary buds' development, which involved proliferation and development of plants from lateral meristems (Ngezahayo and Liu, 2014). The shoots then can be cut off and subcultured to a rooting medium (Figure 5). This propagation is most accurate since this method involves the initiation and development of new shoots from preexisting meristems rather than cell dedifferentiation of already differentiated cells (Grafi et al., 2011).

**Figure 6:**
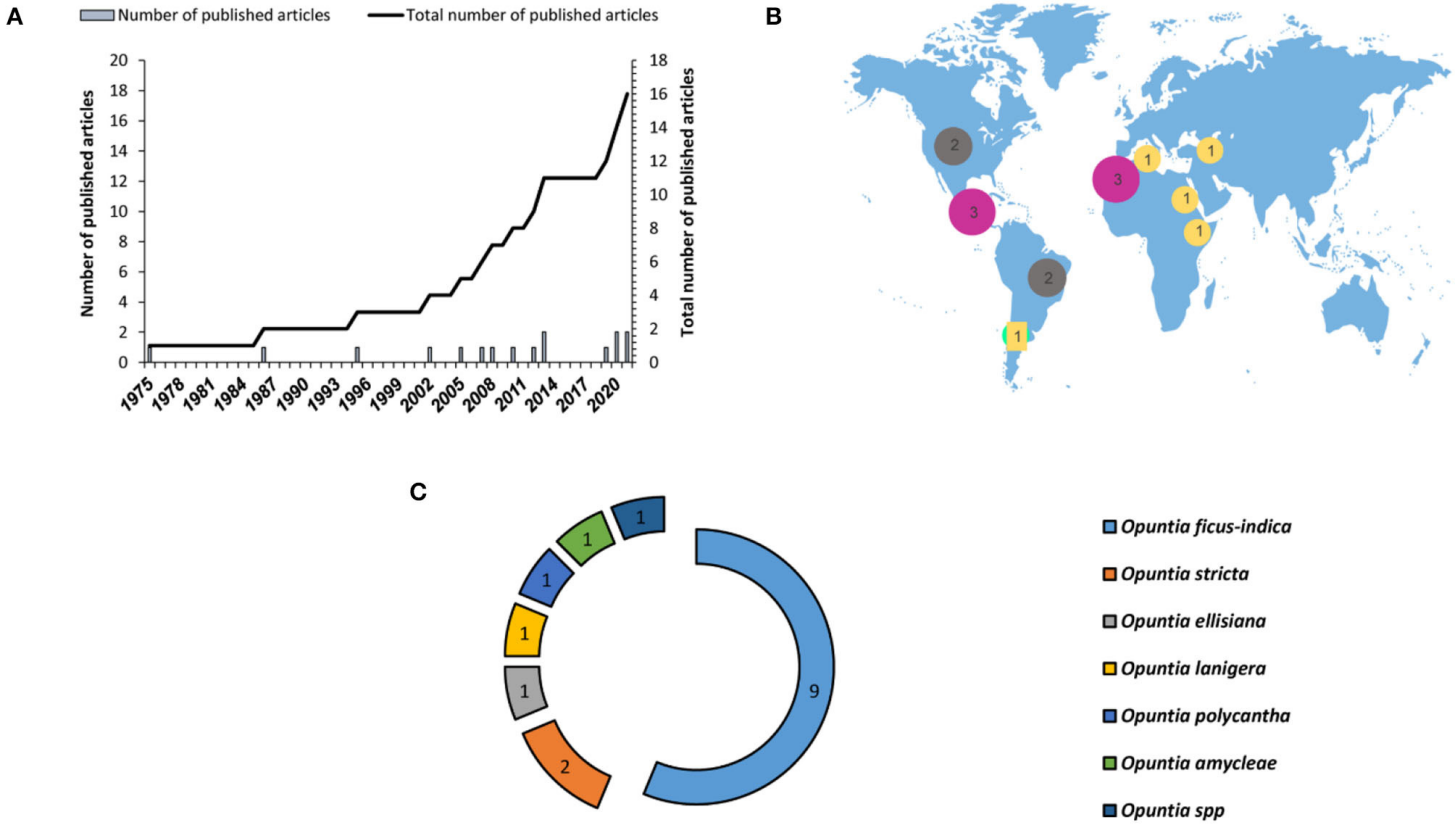
Number of published research articles on *Opuntia* micropropagation through axillary buds' proliferation. **(A)** Number of published articles worldwide during the last three decades, **(B)** geographical distribution of the published research articles, and **(C)** number of research studies depending on the plant species.

Despite several advantages offered by this method of micropropagation, there is limited research regarding the application of this method in *Opuntia* genus. *In vitro* shoot organogenesis can be applied successfully for *Opuntia* micropropagation, giving a high percentage of regeneration. Figure 6 demonstrates the research gaps in the use of this propagation method for prickly pear species. Till now, a limited number of studies (16 published articles from 1975 to 2021) were carried out on *Opuntia* species *in vitro* multiplication through axillary buds' sprouting and 65% of these studies were carried out on *O. ficus*-*indica* (Figure 6). Regarding geographical location, the previous works on axillary buds' proliferation were mainly conducted in Mexico and North African countries and the number of published works remains low. The success of micropropagation through axillary buds' proliferation relies on many factors, such as the explant type, age, and origin of the *Opuntia* plant, the mineral composition of the culture medium, and the addition of plant growth regulators in the culture medium and their concentrations.

### Explant Type

Micropropagation through axillary buds' sprouting can be initiated using any portion of plant tissue comprising areoles. As shown in Table 3, the lower shoot induction or multiplication efficiencies range from 2.0 to 2.8 shoots by a single explant, while the highest recorded was nearby 26.5 shoots per explant in one single cycle. However, if the explants are maintained on a cytokinin-enriched medium, areoles of newly developed shoots can serve as secondary explants from which new shoots can be developed. Therefore, it is possible to deploy the production of *in vitro* plants on large scale. For *O. ficus*-*indica*, the *in vitro* deployment estimated was 1,294 plants from a single explant in four cycles per year and has a potential of 456,976 plants production in a year (Rodriguez and Ramirez-Pantoja, 2020). Explants' age and origin also determine the success of this propagation method. The use of juvenile explants was proven to be highly efficient for *in vitro* propagation of *Opuntia* species. Bougdaoua and El Mtili (2020) had recently established a reliable protocol for *Opuntia ficus-indica* propagation using seedlings (20 mm length) using lower amounts of cytokinin (0.5 mg/L BA). Further, micropropagation efficiency can also be impacted by the explant position or orientation. For example, in *O. lanigera*, the vertical orientation (4.98 shoots per explant) developed more shoots per explant than in a horizontal orientation (3.69 shoots per explant) (Estrada-Luna et al., 2008). However, for *O. amyclaea*, the number of new shoots produced per explant was higher with explants oriented horizontally (Escobar et al., 1986).

**Table 3:** Overview of the media culture and hormonal composition allowing the best responses for shoot induction, multiplication, and rooting reported in previous studies on *Opuntia* micropropagation through axillary buds' sprouting.

Species*	**Shoot induction and multiplication**		**Shoot elongation**		**Shoot rooting**		**References**
	**Hormonal composition giving the best results**	**Number of shoots per explant/multiplication rate**	**Hormonal composition giving the best results**	**Average shoot length**	**Hormonal composition giving the best results**	**Rooting percentage**	
*O. amyclaea*	2.25 mg/L BA	2.0	N/A	N/A	0.1 mg/L IBA	100%	Escobar et al., 1986
*O. ellisiana*	2.25 mg/L BA, 2 mg/L IBA	100%	2.25 mg/L BA, 2 mg/L IBA	10.2 mm	5 mg/L IBA	100%	Juárez and Passera, 2002
*O. lanigera*	5 mg/L BA	5.979	DAP	14.056 mm	N/A	N/A	Estrada-Luna et al., 2008
*O. ficus-indica*	5 mg/L BA	15.0	0.1 mg/L BA	9.39 mm	0.5 mg/L IBA, 0.5 mg/L IAA	100%	El Finti et al., 2010
*O. ficus-indica*	5 mg/L BA	26.5	N/A	N/A	Free hormones, 0.5 mg/L IAA	100%	Khalafalla et al., 2007
*O. ficus-indica*	2 mg/L BA, 0.1 mg/L NAA	8.6	1 mg/L BA	15.2 mm	1 mg/L or 2 mg/L IBA	N/A	Mohamed-Yasseen et al., 1995
*Opuntia ficus-indica*	0.5 mg/L BA	12.28	1 mg/L BA	14.86 mm	N/A	N/A	Bougdaoua and El Mtili, 2020
*O. ficus-indica*	0.5 mg/L BA	100%	0.5 mg/L BA	50 mm	1 mg/L IBA	N/A	Zoghlami et al., 2012
*O. ficus-indica*	5 mg/L BA, 1 mg/L IAA 5 mg/L BA, 0.25 mg/L IAA	2.8	5 mg/L BA, 0.5 mg/ L IAA 5 mg/L BA, 0.25 mg/L NAA	5.3–7.13 mm	–	100%	Ghaffari et al., 2013
*O. ficus-indica*	2 mg/L BA, 0.2 mg/L NAA	5.57	6 mg/L Kin, 2 mg/L IAA	48.80 mm	6 mg/L Kin, 2 mg/L IAA	N/A	Rodriguez and Ramirez-Pantoja, 2020
*O. ficus-indica*	1 mg/L or 2 mg/L Kin	95.75	0.5 mg/L Kin	47 mm	IBA and IAA concentrations	100%	Gebretsadik et al., 2013
*O. ficus-indica*	0.5 mg/L BA	9.6	N/A	N/A	1 mg/L IBA	100 %	García-Saucedo et al., 2005
*O. polyacantha*	2.25 mg/L BA	100%	N/A	N/A	1.8 mg/L NAA	100%	Mauseth and Halperin, 1975
*O. spp*.	5 mg/L BA, 0.5 mg/L NAA	71.74%	N/A	N/A	0.5 mg/L NAA	N/A	Mabrouk et al., 2021
*O. stricta*	1.5 mg/L BA, 0.0625 mg/L NAA	2.05–3.06	1.5 mg/ L BA, 0.0625 mg/L NAA	>20 mm	N/A	N/A	Ferreira et al., 2021
*O. stricta*	0.5 mg/L BA, 0.1 mg/L IAA	14.0	0.26 mg/L mT, 0.1 mg/L IAA	23 mm	0.5 mg/L BA, 0.1 mg/L IAA	100%	de Souza et al., 2019

* *All experiments were performed using MS media*.

### Aseptic Culture Establishment

The success of micropropagation through axillary buds' proliferation also depends on the disinfection procedure. The presence of glochids and thorns makes the disinfection process and the application of aseptic cultures extremely challenging. Earlier, sodium or calcium hypochlorite has been used for the surface sterilization of *Opuntia* species. Mabrouk et al. (2021) suggested a combination of 4.23% of calcium hypochlorite and 0.4% Tween 20 for 10 min as an efficient procedure for *Opuntia spp* aseptic culture establishment. Juárez and Passera (2002) succeeded in *O. ellisiana* surface sterilization by soaking cladodes in 20% sodium hypochlorite–Tween 80 for 10 min, followed by immersion in 1% benzalkonium chloride for 30 min. Two-step disinfection of *O. amyclaea* cladodes was performed by soaking in 70% alcohol for 1 min, followed by an active immersion in 60% calcium chloride for 10 min (Escobar et al., 1986). For *O. lanigera*, a spiny ornamental cactus, aseptic cultures have been established using 70% of ethanol for 5 min, followed by cladodes' deep immersion in a commercial bleach solution (6% of sodium hypochlorite) for 30 min (Estrada-Luna et al., 2008). However, the sodium hypochlorite–ethanol combination was not efficient for the disinfection of *O. robusta* and *O. ficus-indica* cladodes' surfaces due to the wide range of pathogens encountered in spines, areolas, and glochids. This contamination issue was overcome using biocide solution (García-Saucedo et al., 2005) or oxygenated water (30%) (Rodriguez and Ramirez-Pantoja, 2020) in combination with ethanol and commercial bleach.

### Culture Media Formulation

Culture media formulation usually designates salt, vitamins, sugar, and growth regulators. Numerous culture media formulations have been used for plant micropropagation through axillary bud proliferation. The few earlier studies conducted on *Opuntia* genus have used MS medium (Estrada-Luna et al., 2008; Angulo-Bejarano and Paredes-López, 2011; Zoghlami et al., 2012; Ghaffari et al., 2013; Vidican, 2015; Ferreira et al., 2021; Mabrouk et al., 2021). It is widely admitted that salt formulation plays a crucial role in the prickly pear shoot initiation and development. Potassium nitrate, a major salt constituent of MS medium, is required for areoles' activation process. Potassium nitrate triggers the induction and the differentiation process of the aerial parts, and it plays the role of osmoregulator. Ferreira et al. (2021) induced shoot formation in *O. stricta* using a commercial nitrogen fertilizer instead of potassium nitrate and showed that MS medium can be amended with 1 g/L of a potassium nitrate-based fertilizer (Ferreira et al., 2021). Clayton et al. (1990) suggested the potential of other media formulations. These authors tested the efficiency of five different mineral formulations of culture medium (L2, Gamborg B5 (B5), SH, MS, and half-strength MS) on the number of new shoots produced by explant. They found that L2 medium was the most effective, yielding 5.5 axillary shoots per explant on average, while no significant differences in axillary shoot proliferation have been recorded between the four remaining mediums. Clayton et al. (1990) linked this great explant response with the chemical composition of the media culture, mainly high magnesium and calcium ions concentrations. In contrast, the use of a half-strength MS medium yielded the lowest axillary bud proliferation due to low salts concentration. Given the high axillary buds' proliferation obtained using the L2 medium for many cacti species including endangered ones, it could be interesting to explore the potential of this medium for *in vitro* shoot organogenesis.

### Plant Growth Regulators

As mentioned above, the presence of specific plant growth regulators is necessary to stimulate the development of axillary buds. The latter can be assessed by removing the inhibitory effect exerted by the apical meristem on lateral or axillary buds (Bredmose and Costes, 2017). Apical dominance suppression can be triggered by cytokinin application. It was previously established that cytokinin removes apical dominance and activation of lateral buds, and *Opuntia* genus is not excluded from this rule. The use of cytokinin at high or moderate concentrations (1–5 mg/L BA or 1–2 mg/L Kin) was efficient to induce areoles sprouting (Table 3). For *O. ficus-indica*, the presence of BA is mandatory (Khalafalla et al., 2007). These authors found that MS medium supplementation with 5 mg/L BA yielded an average of 26.5 shoots per explant. Axillary buds' activation was also BA-dependent for *O. polyacantha*, at a concentration of 2.25 mg/L (Mauseth and Halperin, 1975). Even though the presence of cytokinin seems to be sufficient for areoles activation, several studies emphasize that the presence of auxin at low concentrations (0.0625 mg/L−0.5 mg/L NAA or 0.1 μM−1 mg/L IAA) in combination with moderate or high amounts of BA increases axillary shoot production in some prickly pear species (Table 3). For *O. ellisiana*, areoles activation can exclusively be stimulated using either a combination of zeatin and NAA (Clayton et al., 1990) or BA and IBA (Juárez and Passera, 2002). Shoot proliferation in *O. ficus*-*indica* was positively correlated with BA concentrations only, while no positive effect of NAA was reported during this study (El Finti et al., 2012). For other species, higher BA amounts inhibit axillary shoot development (Mohamed-Yasseen et al., 1995). Mabrouk et al. (2021) recently reported that the absence of BA reduced the caulogenesis to 0%, while it reached 100% in the presence of moderately high amounts of BA (4–6 mg/L). Media culture supplementation with both BA and Kin has an inhibitive effect on axillary shoots formation, which clearly demonstrates that the balance between endogenous and exogenous cytokinins is crucial for *in vitro* axillary buds' activation as previously reported by Pérez-Molphe-Balch et al. (2015).

Shoot elongation can be easily triggered by the presence of cytokinin alone or in combination with low amounts of auxins. The presence of BA alone amended is sufficient to promote shoot elongation in *O. ficus*-*indica*. The presence of BA at low concentrations (0.1–0.5 mg/L) promoted the length of regenerated shoots to a maximum of 9.39–50 mm (El Finti et al., 2010; Zoghlami et al., 2012). Higher BA concentrations (1–5 mg/L) less promoted shoot elongation in *O. ficus-indica*. Ghaffari et al. (2013) recorded an average of 3 mm of shoot elongation in the presence of 5 mg/L BA. Meanwhile, the combination of BA (5 mg/L) with 0.25 mg/L of NAA significantly increased shoot length (by 7.13 mm) in *O. ficus-indica*. The increase in shoot length was attributed to the presence of auxins, widely known for their stimulatory role in cell division and elongation (Rodriguez and Ramirez-Pantoja, 2020). Shoot elongation can also be stimulated in the presence of low Kin concentrations alone (Gebretsadik et al., 2013) or by the combination of high Kin and moderate IAA amounts (Rodriguez and Ramirez-Pantoja, 2020). Beneficial effects of cytokinin–auxin on shoot elongation have also been reported in other *Opuntia* species. Ferreira et al. (2021) recorded the highest *O. stricta* shoot length values while cultivating newly formed shoots in the presence of 1.5 mg/L of BA and 0.0625 mg/L of NAA. Thus, the combination of low-to-moderate concentrations of cytokinins and low auxin amounts is required for a great shoot elongation for *Opuntia* genus. Besides plant growth regulator concentrations, shoot elongation was found to be species-dependent (Table 3) and sprout lengths are highly variable (Table 3). For *O. ficus-indica*, the best PGR treatments allowed the elongation of newly formed shoots to reach a maximum of 50 mm (Zoghlami et al., 2012), while the highest shoot elongation was 60% and 80% lower for *O. stricta* and *O. ellisiana* shoots (i.e., 20 and 10.2 mm, respectively) (Juárez and Passera, 2002; Ferreira et al., 2021).

The final step of micropropagation through axillary buds' proliferation is related to adventitious root formation or rhizogenesis. This step is usually initiated when newly formed shoots reached a specific length. For cacti, rooting can start once the length of the produced shoots is >5 mm (Lema-Rumińska and Kulus, 2014). Rooting induction medium commonly comprises auxin sources. IBA and IAA or NAA are widely used to trigger rhizogenesis in *Opuntia* species (Escobar et al., 1986; Mohamed-Yasseen et al., 1995; El Finti et al., 2010; Zoghlami et al., 2012; Mabrouk et al., 2021). One hundred percent of rooting has been recorded in *O. ficus-indica, O. robusta, O. amyclaea*, and *Opuntia spp*. in the presence of low-to-moderate amounts of IBA or IAA or NAA (0.5–2 mg/L) (Table 3). However, in *O. ellisiana*, rhizogenesis can only be triggered in the presence of high auxin amounts (5 mg/L IBA) (Juárez and Passera, 2002). In some cases, the presence of auxin in the media culture can inhibit root formation due to the high amounts of endogenous auxins. Thus, rooting in *O. ficus-indica* can occur spontaneously without PGRs (Khalafalla et al., 2007; Ghaffari et al., 2013).

## Conclusion

An appropriate micropropagation method is required for the mass propagation for the commercial purpose, for the conservation of endangered species, and for ecosystem conservation and restoration. Conventional propagation methods (sexual propagation through seeds, vegetative propagation by cuttings, and grafting) have been used for centuries to propagate cacti species with great interest. However, these traditional techniques cannot be implemented to mass propagate and the scale-up production of commercial species given the limited number of plants that appears to be scarce with market needs. In this context, micropropagation has been proved as a powerful tool in both experimental biotechnology (regeneration and conservation biology) and horticultural production aiming food and fruit production and for ornamental and medicinal purposes. We found that micropropagation through axillary buds' proliferation has been adopted for several cacti genera, including *Mammillaria, Echinocereus, Hylocereus*, and *Ariocarpus;* however, less research has been conducted on the *Opuntia* genus. Based on this review, the following prospect can be proposed:

First, one of the factors affecting the success of micropropagation is related to the removal of fungal and bacterial contaminants from plant material surfaces. Despite the wide range of disinfection procedures (cited in section Fungal and Bacterial Contamination) that have been developed, the success of these procedures is mostly dependent on the species, the nature of plant material, and its growing conditions. Significant work has to be made to establish a reliable protocol for cacti surface sterilization. In the end, the developed method should allow the removal of or the reduction in fungal and bacterial infections without affecting explants.

Second, micropropagation through axillary buds' proliferation can supply a great asset to meet market needs for human uses (fruits) and animal forage. However, the success of this propagation method depends on the growth conditions (media culture composition). The previous studies were focused on the use of one single mineral culture formulation, that is, MS medium in combination with plant growth regulators, such as BA, NAA, IAA, and IBA. Better results can be achieved using other media culture formulations as demonstrated by Clayton et al. (1990) in other cacti species. Therefore, future research is required on testing the impact of the mineral formulation of the culture medium on the regeneration process.

Third, the overexploitation and the cochineal infestation of *Opuntia* species limit considerably the maintenance and the preservation of these species in their natural areas, one of the solutions that can be improved brought to explore genetic diversity. Some of the presented methods of propagation can contribute to and/or increase genetic diversity. Those methods should be used to develop tolerant/resistant genotypes and ecotypes.

Last, *Opuntia* and other cacti display great nutritional and medicinal properties conferred by the presence of bioactive compounds. *In vitro* culture techniques can be used to increase the production of these high-value chemical compounds. Cell dedifferentiation (callogenesis) and organogenesis can offer a promising pathway to produce high amounts of plant-derived secondary metabolites. Cell suspensions (derived from cell dedifferentiation) should be used for the large-scale production of these bioactive molecules (in bioreactors) to meet the increasing demand for plant-derived secondary metabolites for human health.

## Author Contributions

SB: conceptualization, methodology, investigation, data analysis, interpretation, writing–original draft, and review and editing. EE, LK, KD, HB, and MS: methodology and review and editing. YE: conceptualization, methodology, validation, review and editing, project administration, and supervision. All authors have read and approved the final version of the document.

## Funding

This research was funded by OCP Phosboucraâ Foundation, Laâyoune, Grant No. PR008.

## Conflict of Interest

The authors declare that the research was conducted in the absence of any commercial or financial relationships that could be construed as a potential conflict of interest.

## Publisher's Note

All claims expressed in this article are solely those of the authors and do not necessarily represent those of their affiliated organizations, or those of the publisher, the editors and the reviewers. Any product that may be evaluated in this article, or claim that may be made by its manufacturer, is not guaranteed or endorsed by the publisher.
